# Tailoring FXR Modulators for Intestinal Specificity: Recent Progress and Insights

**DOI:** 10.3390/molecules29092022

**Published:** 2024-04-27

**Authors:** Amanda Morrison, Bahaa Elgendy

**Affiliations:** 1 Center for Clinical Pharmacology, Washington University School of Medicine and University of Health Sciences and Pharmacy, St. Louis, MO 63110, USA; amanda.morrison@uhsp.edu; 2 Department of Anesthesiology, Washington University School of Medicine in St. Louis, St. Louis, MO 63110, USA

**Keywords:** farnesoid X receptor (FXR), intestinal selectivity, bile acid synthesis, metabolic diseases, drug design

## Abstract

While FXR has shown promise in regulating bile acid synthesis and maintaining glucose and lipid homeostasis, undesired side effects have been observed in clinical trials. To address this issue, the development of intestinally restricted FXR modulators has gained attention as a new avenue for drug design with the potential for safer systematic effects. Our review examines all currently known intestinally restricted FXR ligands and provides insights into the steps taken to enhance intestinal selectivity.

## 1. Introduction

The Farnesoid X Receptor (FXR) is a nuclear receptor that is activated by endogenous bile acids (BAs) [1]. BAs are amphipathic molecules that help to solubilize fats in the intestine after a meal [2]. High levels of BAs activate FXR, which then initiates a feedback loop that reduces cholesterol and BA synthesis [1,2]. FXR is found in higher amounts in tissues exposed to high levels of bile acids, including the liver, small intestine, duodenum, gall bladder, colon, and rectum. The liver has the highest concentration and the rectum has the lowest [3]. FXR is expressed in a variety of tissues not involved in bile acid sensing, including the urinary bladder, steroidogenic tissues, immune cells, brain, cardiovascular system, and adipose tissue [3].

**Figure 1:**
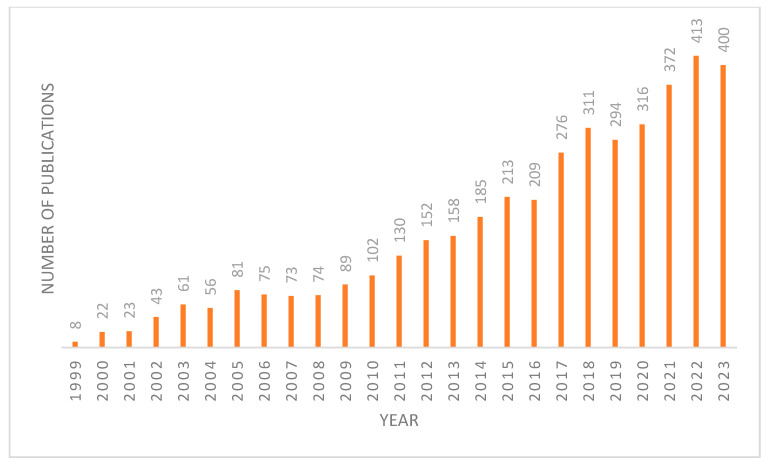
Number of articles published on FXR each year since 1999 from Pubmed database.

FXR plays a crucial role in metabolism due to its enterohepatic activities, highlighting its significance as a metabolic regulator. This has spurred increased interest in targeting FXR, evidenced by the growing number of publications on this topic in recent years (Figure 1). FXR acts as a homeostat of three classes of nutrients—fats, sugars, and proteins—by regulating bile acid homeostasis, lipoprotein, and glucose metabolism [4]. When activated, FXR has three ways of regulating bile acid levels in the body [5]. The first is by increasing the expression of a protein called a small heterodimer partner (SHP) [1]. SHP is a transcriptional repressor that inhibits the expression of CYP7A1, the rate-limiting enzyme in BA synthesis. The second is by stimulating the expression of fibroblast growth factor 15/19 (FGF15/19). Upon FXR activation, it binds to the second intron of FGF15, which then binds to fibroblast growth factor receptor 4 (FGFR4) on the surface of hepatocytes. This activates the C-Jun N-terminal kinase (JNK) pathway, which inhibits the production of bile acids [6]. Lastly, FXR directly activates the expression of the bile salt export pump (BSEP), which enhances the excretion of BAs and cholesterol into the feces [1]. This feedback loop results in a reduction in the bile acid pool, as bile acid production decreases and excretion from the liver increases [7].

Activation of FXR in lipid metabolism leads to suppression of the sterol response element binding protein (SREBP) transcription factors. This, in turn, downregulates the expression of cholesterol and fatty acid synthesis genes fatty acid synthase (FAS) and Acetyl-CoA Carboxylase (ACC), as well as lipoprotein lipase secretion genes apolipoprotein C-II/III (APOC2/3) and apolipoprotein E (APOE). Meanwhile, the expression of the LDL receptor is increased. The LDL receptor enhances lipoprotein remnant reuptake and can reduce the expression of microsomal triglyceride transfer protein, which regulates the assembly of very-low-density lipoproteins (VLDL) [1,8]. Additionally, FXR activation promotes the clearance of circulating triglycerides by the very-low-density lipoproteins receptor (VLDL-R) and the expression of Syndecan-1. It also represses APOC3, which increases lipoprotein lipase activity, and may directly enhance the expression of phosphoenol-pyruvate carboxykinase 1 (PEPCK1), a rate-limiting enzyme essential for maintaining blood glucose balance [8].

An unexpected link between FXR and hepatic glucose metabolism has been found in recent years. When the body is in a fed state, glucose can be converted to glycogen through glycogenesis, while in a fasting state, glucose is converted to ATP via glycolysis or gluconeogenesis [6]. Studies on FXR-null mice showed that they suffered from mild glucose intolerance and insulin insensitivity [9,10]. In addition, activation of hepatic FXR has been found to decrease plasma glucose levels and downregulate the gluconeogenic pathway. When FXR is activated in the liver, it can suppress three different gluconeogenic enzymes through SHP: PEPCK, fructose1,6-bis phosphatase (FBP1), and glucose-6-phosphatase (G6Pase) [9]. FXR activity has also been shown to improve insulin sensitivity in various tissues by reducing triglycerides and free fatty acids. Activation of FXR has also been associated with inducing glycogen synthesis; it has been shown in cell studies that FGF19 stimulates glucose uptake and has insulin-independent effects on glycogen synthesis [10]. Finally, it has been suggested that FXR activation can result in insulin substrate receptor 1 (IRS-1) tyrosine phosphorylation in both the liver and adipose tissue, which could play a major role in insulin resistance [11]. These various studies have shown how FXR activation can be used to improve glycemic parameters.

**Figure 2:**
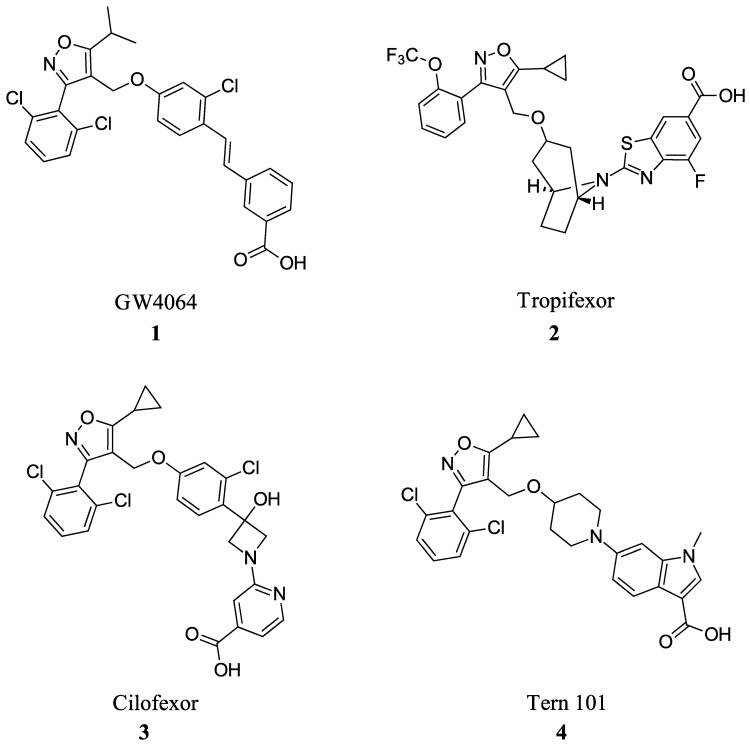
FXR agonists GW4064 (**1**), Tropifexor(**2**), Cilofexor (**3**), and Tern 101 (**4**).

Many FXR agonists fail clinical trials due to side effects such as dyslipidemia, pruritus, and liver decomposition [1]. Interestingly, all non-steroidal agonists were derived from GW4046 (Figure 1). GW4064 (**1**) is the first non-bile acid FXR agonist and has remained the main template for most non-steroidal agonists since its discovery (Figure 2). Although GW4064 (**1**) is UV sensitive and potentially toxic, several derivatives of GW4064 (**1**) have made it to clinical trials such as Tropifexor (**2**), Cilofexor (**3**), and Tern-101 (**4**) (Figure 2) [12]. Although these compounds show promise in treating NASH, their advancement has been hindered by the presence of unwanted side effects like pruritus and dyslipidemia. This leaves a need to design a novel FXR agonist with the potential to eliminate harmful side effects.

### 1.1. FXR in the Gut

During digestion, cholecystokinin triggers gallbladder contraction and the delivery of bile to the small intestine. BAs facilitate the production of fibroblast growth factor FGF15/19 induced by FXR. This growth factor then binds to the liver’s FGFR4/β-Klotho complex. This causes CYP7A1 suppression in a synergistic manner with FXR–SHP-dependent inhibition of CYP7A1 in the liver [9,13]. In enterocytes, BAs are shuttled through membranes by intestinal bile acid binding protein (IBABP) and secreted into the liver by organic solute transporter OSTα/β [14]. BAs are also reabsorbed into the intestine through the apical sodium-dependent bile acid transporter (ASBT). FXR downregulates ASBT expression and upregulates IBABP and OSTα/β, promoting the transport of BAs to the liver [9,15].

Gut microbiota plays an important role in FXR-mediated signaling and metabolism. Commensal bacteria in the intestine are involved in the transformation of primary bile acids into secondary bile acids through bile salt hydrolase (BHS) activity by conjugation with glycine or taurine [16]. These secondary bile acids, such as tauroβ-muricholic acid (T-βMCA) and deoxycholic acid, are potent endogenous ligands for FXR [16]. Activation of intestinal FXR by BAs influences the composition of the gut microbiota, establishing a feedback loop between the host and microbiota. Given the interplay between BAs, FXR, and gut microbiota, it is increasingly recognized that targeting intestinal FXR can be accomplished through gut microbiota. Caffeic acid phenethyl ester (CAPE) has been found to alleviate obesity-related steatosis through microbiota by increasing endogenous antagonist T-βMCA [17]. In addition, Metformin has been found to improve insulin resistance through a B. fragilis–GUDCA–intestinal FXR axis [18]. A better understanding of these interactions could lead to the development of new microbiota-directed therapies for metabolic diseases via FXR.

### 1.2. Importance of Intestinal Modulation

Systemic activation of FXR has been shown to lead to adverse side effects in clinical trials, such as pruritus, imbalance in cholesterol homeostasis, gastrointestinal effects, and an increased risk for liver decomposition [19]. To address these concerns, researchers have focused on developing intestinally selective FXR agonists. The first attempt was the development of Fexaramine (Fex) (**20**), which induced effects on enteric FGF19 without activating any target genes in the liver [20].

Intestinal FXR is essential in mitigating several disease states. For example, high levels of intestinal BAs were found to increase the risk of colorectal cancer (CRC) [21]. Changes in BA profiles lead to malignant transformations in Lgr5-expressing (Lgr5+) cancer stem cells and promote adenoma-to-adenocarcinoma progression. The mechanism behind this is that BAs that antagonize intestinal FXR function, such as T-βMCA and DCA, induce proliferation and DNA damage in Lgr5+ cells. On the other hand, selective activation of intestinal FXR can limit abnormal Lgr5+ cell growth and reduce the progression of CRC. These results suggests that selective intestinal FXR activation could be a potential therapeutic target for CRC [22]. In metabolic diseases, it has been suggested that intestinal agonists alter the BA pool to contain higher levels of lithocholic acid, causing downstream activation of the G protein-coupled BA receptor (TGR5), improving energy expenditure as well as browning of white adipose tissue [20]. In glucose metabolism, intestinal FXR antagonism in L-cells has been shown to increase glucagon-like peptide-1 (GLP-1) production, inducing insulin secretion. Therefore, intestinal FXR antagonism has the potential to improve glucose metabolism and treat type 2 diabetes [23].

## 2. FXR Modulators

### 2.1. Steroidal Based Modulators

**Figure 3:**
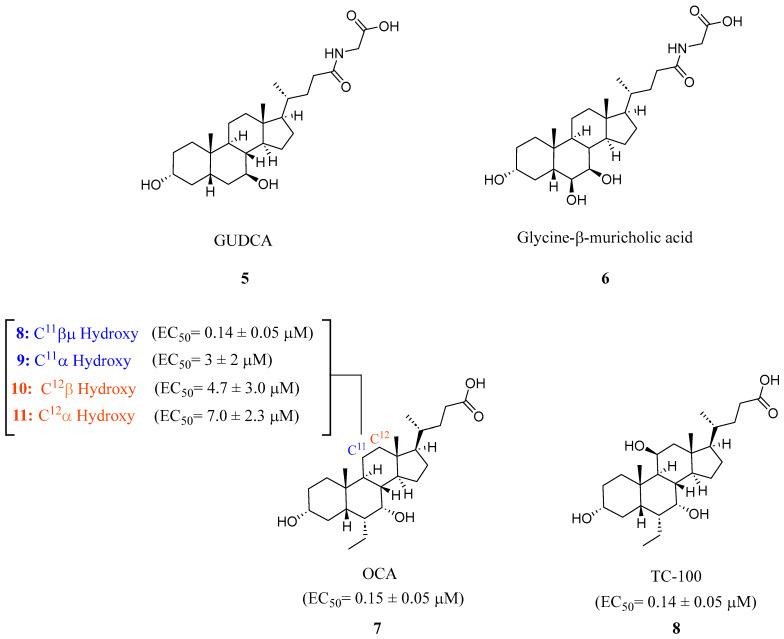
Steroidal FXR ligands GUDCA (**5**), Gly-MCA (**6**), OCA (**7**), and TC-100 (**9**).

Steroidal FXR ligands have been available for a long time as they utilize the scaffolds from FXR’s natural ligands, BAs. The most potent endogenous ligand of FXR is chenodeoxycholic acid (CDCA), which led to the development of obeticholic acid (OCA) (**7**) (Figure 3) [24]. OCA (**7**) has since been the first FXR ligand to enter clinical trials and has been approved for the treatment of primary biliary cholangitis (PBC) since 2016 [24]. However, it is important to note that OCA’s new drug application was denied due to its moderate efficacy and considerable risk of side effects such as pruritis, which occurred in 56% of patients receiving 25 mg of OCA daily [25]. Since the discovery of OCA (**7**), many other steroidal FXR modulators have been of great interest for potential drug development.

In recent studies, it has been discovered that glycoursodeoxycholic acid (GUDCA) (**5**) is an endogenous BA antagonist of FXR (Figure 3) [18]. GUDCA levels have been reported to be regulated inversely through levels of the bacteria B. fragilis and its BSH activity [18]. It was found that in a TR-FRET FXR coactivator assay, GUDCA (**5**) antagonized intestinal CDCA activation of FXR (IC_50_ = 77.2 μM) while inhibiting CDCA-induced FGF19 and SHP expression in a luciferase reporter gene assay [18].

In a study on mice fed a high-fat diet (HFD) and given GUDCA (**5**) orally for a week, it was found that GUDCA (**5**) specifically affected the intestinal FXR signaling pathway. Different doses of GUDCA (**5**) (5, 10, and 50 mg/kg/d) were administered, and the results demonstrated an impact solely on the intestinal FXR signaling. GUDCA (**5**) treatment improved glucose tolerance, insulin resistance, and attenuated weight gain in HFD wild-type mice but not in HFD mice lacking intestinal FXR. GUDCA (**5**) induced thermogenic gene expression in wild-type mice but had no further induction on Fxr^∆IE^ mice [18]. To examine the effect on metabolic disorders, GUDCA (**5**) was administered (50 mg/kg/d) for 4 weeks and showed therapeutic effects in reversing metabolic disorders in established HFD mice through intestinal FXR antagonism, increased metabolic rate, and GLP1 production [18].

The steroidal FXR antagonist glycine-β-muricholic acid (Gly-MCA) (**6**) was identified through computational modeling as a potent inhibitor of intestinal but not hepatic FXR (Figure 3). This compound was rationally designed based on the structural scaffold of the closely related antagonist T-β-MCA (TCA). Notably, Gly-MCA (**6**) demonstrates favorable pharmacokinetic properties, such as oral bioavailability and resistance to hydrolysis by gut bacterial bile salt hydrolase [26]. In mice with diet-induced obesity or a genetic Lepr mutation (db/db), oral administration of Gly-MCA (**6**) at 10 mg/kg selectively decreased mRNA expression of the FXR target genes SHP and FGF15 in the ileum. It also reduced ceramide levels and expression of genes involved in ceramide metabolism. Gly-MCA (**6**) tissue-selective antagonism holds therapeutic potential for metabolic diseases. While Gly-MCA (**6**) could serve as a lead for developing intestinally restricted FXR antagonists, further studies are still needed to thoroughly evaluate its safety profile in various disease models. Continued medicinal chemistry efforts aimed at optimizing this scaffold may help generate additional candidates for preclinical investigation.

A novel FXR ligand, 3α,7α,11β-trihydroxy-6α-ethyl-5β-cholan-24-oic acid (TC-100) (**8**), was developed with a BA scaffold expanding beyond C6α alterations commonly seen since the development of OCA (**7**) (Figure 3) [27]. To expand on the commonly known C^6^α alterations, various substitutions were made in various locations on known bile acid OCA (**7**) and docked in crystal structure 4QE6. This revealed that hydroxyl-containing derivatives in the C^11^β and C^12^β positions engage in an additional hydrogen bond with Leu284. In an AlphaScreen assay and a homogeneous time-resolved fluorescence (FRET) cell-based assay, it was found that placing a hydroxyl group at C^11^β position (**8**) could be the new hotspot for alteration as it was found to have slightly improved activity toward FXR (EC_50_ = 0.14 μM), higher efficacy than OCA (**7**), and lacked activity at TGR5 (Figure 3) [27].

Preliminary pharmacokinetic studies revealed that intraduodenal (id) administration led to TC-100 (**8**) being quickly secreted in bile in the liver faster than endogenous BAs, making its activation intestinally localized. Although TC-100 (**8**) is less lipophilic than other BAs, its low lipophilicity causes rapid secretion from the liver, reducing hepatic residence time. Using RT-PCR assays in HepG2 cells, it was found that TC-100 (**8**) is equipotent to OCA in modulating intestinal FXR target genes CYP7A1, SHP, and OSTα and was more efficient than OCA in stimulating BSEP [27]. Male C57BL/3 mice with obstructive cholestasis were treated with oral gavage of vehicle (1% methylcellulose), OCA and TC-100 (**8**), for 5 days at a dose of 10 mg/kg/d. The results demonstrated that TC-100 (**8**) significantly induced the expression of FGF15, SHP, and ANG1 genes in the ileum compared to both OCA and vehicle [27]. TC-100 (**8**) treatment led to the repression of CYP7A1 due to the hepatic effect of ileal FGF15. Therefore, it was concluded that intestinal activation of FXR using TC-100 (**8**) stimulated the gut–liver axis and eventually caused FGF15-mediated CYP7A1 repression in the liver.

**Figure 4:**
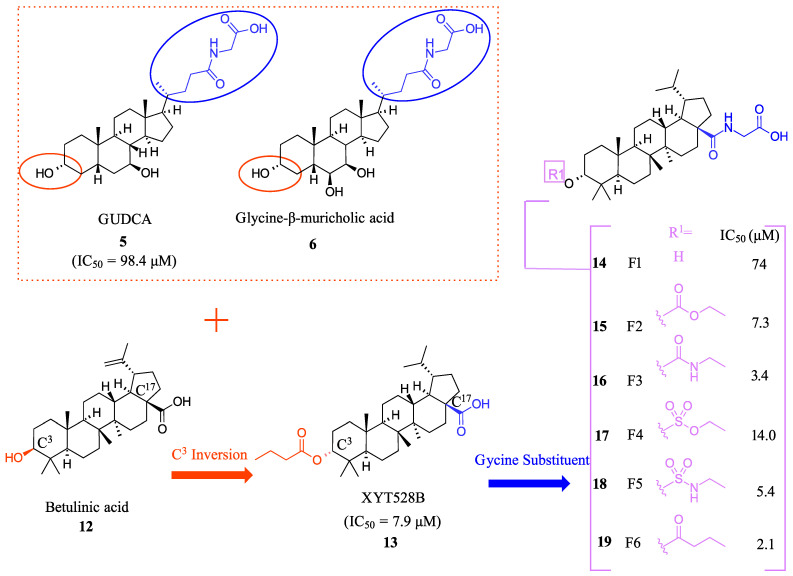
Development of F6 (**19**).

F6 (**19**) was developed as a potent intestine-selective FXR antagonist for the treatment of NASH [28]. Betulinic acid (**12**) was chosen as the starting point for developing **19** since it had previously been reported as an FXR agonist (Figure 4) [29]. In order to further mimic bile acids, the hydroxyl group at the C^3^ position was inverted to the α position and converted to an ester, leading to XYT458B (**13**) (IC_50_ = 7.9 μM) (Figure 4). This compound was found to be a potent TGR5 agonist but was very cytotoxic. Then, inspired by intestinally selective Gly-MCA (**6**) and GUDCA (**5**), a glycine was conjugated to the C^17^-COOH (Figure 4) [28]. This modification resulted in weak antagonistic activity (IC_50_ = 74.0 μM), but the addition of a butyrate group to the hydroxyl at C^3^α- position led to identification of the more potent F6 (**19**) with an IC_50_ of 2.1 μM (Figure 4). Any changes to the glycine group at C^17^ were not tolerated, emphasizing the importance of this group in F6 (**19**) [28]. Molecular modeling has shed light on the importance of the carboxyl group of glycine in bonding, as it forms hydrogen bonds with His460 and Trp482, which make it crucial for binding. F6 (**19**) decreased SHP, FGF15/19, and OSTα in the intestine and upregulated CYP7A1 and CYP8B1 in the liver, indirectly demonstrating how F6 (**19**) upregulates bile acid synthesis [28].

The role of F6 (**19**) in regulating intestinal FXR expression was assessed in isolated epithelial crypts to construct small intestinal (SI) organoids. When SI organoids were exposed to FXR agonist F6 (**19**), the organoid budding was not inhibited as expected. Instead, F6 (19) actually stimulated the growth of the organoid. Moreover, F6 (**19**) blocked the upregulation of FXR, FGF15, and SHP when treated with 10 µM of GW4064 (**1**) and abolished the upregulation of FXR and FGF15 when treated with 50 µM of CDCA [28].

In microbiota-depleted mice, administering F6 (**19**) orally at a dosage of 10 mg/kg resulted in the downregulation of FG15 and SHP that were induced by the agonist taurocholic acid in the ileum while not altering the hepatic FXR signaling. At a dose of 10 mg/kg, F6 (**19**) was more effective than GUDCA (**5**) at 50 mg/kg in inhibiting taurocholic acid-induced genes.

To evaluate the intestinal selectivity of F6 (**19**), it was orally administered at a dosage of 10 mg/kg in mice; then, tissue distribution in the liver, plasma, and intestine was measured. F6 (**19**) was mainly detected in the intestine and was not detected in the liver or plasma [28]. It was found that F6 (**19**) metabolizes quickly into an inactive metabolite F1 (**14**) in the liver and serum, explaining its low liver exposure and showing that F6 (**19**) is a gut-restricted soft drug [28].

In the GAN-diet-induced NASH mouse model, administration of F6 (**19**) (at a dose of 10 mg/kg) resulted in a reduction in weight gain and improvement in insulin sensitivity and glucose tolerance comparable to that observed with GUDCA (**5**) (administered at a dose of 5 mg/kg). Impressively, F6 (**19**) lowered the liver fibrosis marker hydroxyproline and histological evaluation confirmed that F6 (**19**) improved remission of NASH in GAN-diet fed mice. In addition, F6 (**19**) improved mRNA expression related to lipid metabolism, fibrogenesis, and inflammation. In HFMCD diet fed mice, F6 (**19**) (3, 10, and 30 mg/kg) was compared to known agonist OCA (**7**) (30 mg/kg) and known antagonist GUDCA (**5**) (50 mg/kg) regarding its ability to alleviate NASH symptoms. F6 (**19**) significantly lowered alanine aminotransferase (ALT) and aspartate aminotransferase (AST) levels, hepatic triglyceride (TG) concentration, and hydroxyproline levels, and decreased collagen depositions, when compared to these well-known modulators.

The serum ceramide levels were substantially reduced by inhibiting intestinal FXR signaling with F6 (**19**). Lipidomic tests were conducted on the serum of mice with NASH induced by GAN-diet. Results showed that administering F6 (**19**) and GUDCA (**5**) orally for 12 weeks significantly reduced dihydroceramide, glucosylceramide, sphingomyelin, and ceramide levels in the serum. Additionally, the levels of the free fatty acid (FFA), triacylglycerol (TAG), and cholesteryl ester (CE) remained unchanged. These results showed clearly that F6 (**19**) treatment alleviated NASH symptoms via modulating the FXR–ceramide axis [30].

Systemic FXR activation leads to undesirable side effects, while inhibiting hepatic FXR signaling may lead to severe hepatic toxicity. F6 (**19**), on the other hand, has a unique mechanism of action that allows for selective inhibition of FXR signaling in the intestine and activation of hepatic FXR. Unlike other agonists such as Betulinic acid, F6 (**19**) treatment increases both mRNA and protein levels of CYP7A1, which can help reduce pruritus rates in patients with NASH.

### 2.2. Non-Steroidal Based Modulators

#### 2.2.1. Fexaramine and Its Derivatives

Importantly, it should be noted that none of the aforementioned derivatives of Fex (**20**) activated or bound to RXR.

**Figure 5:**
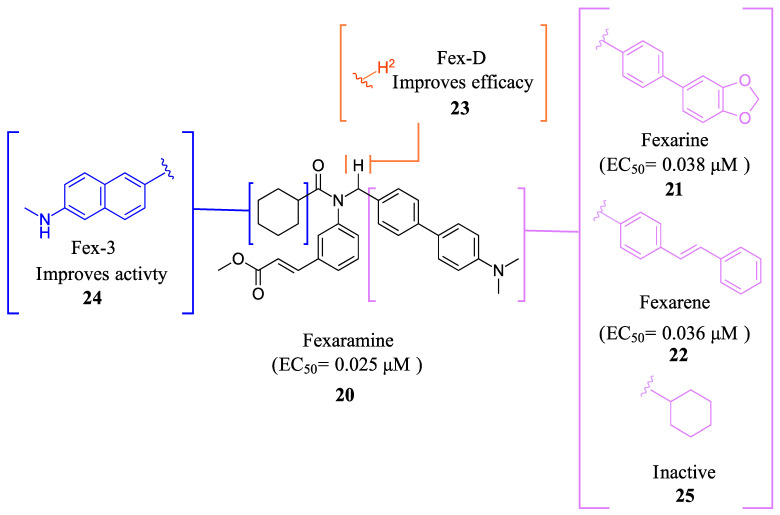
Fex (**20**) and its derivatives.

Fexaramine (Fex) (**20**) (Figure 5) was initially identified as a non-steroidal agonist and was later discovered to have intestinally restrictive properties. Since then, many gut-restricted FXR modulators have been designed based on the structure of Fex (**20**). The discovery of Fex (**20**) was accomplished through high-throughput screening of a benzopyran library against FXR/RXR transfected cells [31]. A number of lead compounds in the prototypical structure were identified and underwent systematic optimization. Consequently, Fex (**20**) (EC_50_ = 0.025 μM) was identified, along with two other derivatives, namely, Fexarine (**21**) (EC_50_ = 0.036 μM) and Fexarene (**22**) (EC_50_ = 0.036 μM), which exhibited outstanding activation of FXR compared to GW4064 (**1**) (EC_50_ ≈ 0.09 μM) (Figure 5) [31]. Importantly, it should be noted that none of the aforementioned derivatives of Fex (**20**) activated or bound to RXR.

It was found that Fex (**20**) displayed coactivator recruitment activation of SRC-1 (EC_50_ = 0.255 μM) in a fluorescence resonance energy transfer-based coactivator assay [31]. To ensure selectivity, a cross-reactivity assay was performed, and all Fex derivatives displayed no transcriptional activity toward other nuclear receptors. A cell-based assay using FXR response elements (minimal TK promoter, TK-ECREx6 promoter, TK-ER8x2 promoter, hI-BABP promoter, hPLTP promoter, or rMRP-2 promoter) demonstrated concentration-dependent activation, with Fex (**20**) performing comparably to GW4064 (**1**) and other Fex (**20**) derivatives performing less efficaciously at 1 µM. In HEPG2 cells, Northern blot assay showed that Fex (**20**) induced target genes SHP, MRP-2, BSEP, and PLTP with efficacy similar to GW4064 (**1**) [31].

In a recent study, molecular docking using the Protein Data Bank (PDB ID: 1OSH) showed that Fex (**20**) engages in specific interactions with distinct regions of FXR’s LBD through two sets of interactions [31]. The first set involves stabilizing Fex’s hexyl ring, outermost benzene ring, and methyl ester moiety by establishing minimal van der Waals contacts with Ile339 and Leu344 through the hexyl group, while hydrophobic amino acids Met369, Phe333, and Phe370 create a surface behind Fex’s nitrogen and benzyl groups [31]. Additionally, Met294 on helix 3, and Leu352 and Ile356 on helix 6, stabilize the aliphatic linker and the methyl ester group through hydrogen bonds [31]. The second set of interactions involves stabilizing the biaryl rings and the dimethyl amine moiety of Fex (**20**) by forming a deep hydrophobic pocket through Phe288, Leu291, Thr292, and Ala295 on helix 3, and Ile361, His451, Met454, Leu455, and Trp458 on helix 11, to accommodate Fex’s biaryl moiety [31].

Additional research has been conducted on Fex (**20**) since its initial discovery, revealing that its effects are restricted to the intestines when taken orally. In vivo studies have shown that administering Fex (**20**) intraperitoneally at a dosage of 100 mg/kg induces the target gene Nr0b2, which encodes for SHP in the liver, kidney, and gut [20]. However, when Fex (**20**) is orally administered, only intestinal Nr0b2 is induced [20]. This finding was further confirmed when oral administration failed to induce FABP6, which encodes for IBABP, and Slc51a, which encodes OSTα and FGF15 in the liver and kidneys [20]. After prolonged intestinal activation of FXR using Fex (**20**), an increase in FGF15 levels in the ileum was observed, while hepatic CYP7A1 was suppressed [20].

Intestine selectivity of Fex (**20**) was found to be beneficial for treating obesity and improving insulin resistance. In mice with diet-induced weight gain, orally administered Fex (**20**) (100 mg/kg) was able to reduce weight gain without causing any intestinal toxicity [20]. Furthermore, mice treated with Fex (**20**) showed improved metabolic and endocrine profiles, with lowered glucose, insulin, leptin, cholesterol, and resistin levels, and increased energy expenditure through oxidative phosphorylation of brown adipose tissue [20]. These findings were confirmed by Fex (**20**) lowering serum lactate levels in DIO mice, shifting body-wide energy metabolism toward a more oxidative state. In white adipose tissue, Fex (**20**) was also found to increase the expression of the beta-3 adrenergic receptor, which induces brown fat-like cells that are associated with resistance to diet-induced obesity and improved glucose metabolism.

It was shown that chronic ethanol intake results in lowering intestinal FXR signaling and increased plasma bile acids. In mice fed with ethanol daily for 8 weeks, treatment with Fex (**20**) reduced ethanol-induced liver injury (as demonstrated by lower plasma ALT levels), steatosis, and decreased ethanol-induced hepatic inflammation [32]. This was achieved without altering the total amount of bacteria in the cecum and maintaining intestinal barrier integrity [32].

FexD (**23**) is the deuterated version of Fex (**20**), which maintains gut-restricted properties while improving in vivo efficacy (Figure 5) [22]. In APCmin/+ mice maintained on ND or HFD (models of adenoma and adenocarcinoma), FexD (**23**) (50 mg/kg daily oral gavage at the time of tumor initiation) decreased fecal bleeding and cancer-induced weight loss in all treatment groups [22]. Histological studies revealed that mice treated with FexD (**23**) had 40% fewer adenomas and 25% fewer adenocarcinomas on average [22]. Additionally, there was a decrease in cell proliferation and improved nuclei morphology. The treatment also reduced systemic inflammation, which was evident by a decrease in spleen weight and serum cytokine levels (IL-17a and IL-10) [22]. APCmin/+ mice on ND showed a 10-week life expectancy increase when treated with FexD at 50 mg/kg/day, indicating its potential in cancer treatment [22].

Additional studies found that mice treated with FexD (**23**) (50 mg/kg/day p.o. for 4 wk) prior to acute dextran sulfate sodium (ADSS) treatment were protected against DSS-induced damage such as changes in serum BA levels and increased intestinal permeability [33]. In addition, FexD (**23**) treatment reduced inflammatory response indicated by spleen size, reduced levels of proinflammatory cytokines IL17 and IL6, and reduced weight loss [33]. FXR is an important regulator of a particular type of immune cell called innate lymphoid cells (ILCs). These cells play a crucial role in managing gut immunity by releasing cytokines. Recent studies have revealed that FexD (**23**) can modulate gut cytokines by affecting the ILCs. FexD (**23**) treatment leads to a three-fold decrease in ILC3 proportions, which results in decreased cytokines such as IL17F and IL33 in ILC3 clusters. This demonstrates that FexD (**23**) can inhibit the development and function of ILCs [33].

Fex-3 (**24**) was designed following the discovery of Fex (**20**) to improve its intestinal selectivity (Figure 5) [34]. Structure-Activity relationship studies of Fex (**20**) showed that the biphenyl group was important for the agonistic activity because, when replaced with a cyclohexyl, no agonistic effect was observed in rat primary hepatocytes [34]. Fex-3 (**24**) maintains the biphenyl moiety but replaces Fex’s cyclohexyl group with methylamine naphthalene (Figure 5). In Caco-2 and HepG-2 cells, Fex-3 (**24**) induced expression of BESP and SHP, and inhibited CYP7A1 more than Fex (**20**). In addition, Fex-3 (**24**) accumulated nearly the same as Fex (**20**) in the intestine while being more easily absorbed into intestinal cells, indicating that Fex-3 (**24**) is more intestinally restricted than Fex (**20**) [34]. To further evaluate the effect of both Fex (**20**) and Fex-3 (**24**) in vivo, studies on mice following intragastric administration of both Fex (**20**) and Fex-3 (**24**) at 100 mg/kg followed by sacrificing the animal after 1 h showed that Fex-3 (**24**) was accumulated only in the ileum and at a higher concentration than Fex (**20**) [34]. On the contrary, Fex (**20**) was detected in appreciable concentrations in the jejunum and in the liver as well.

The observed intestinal selectivity of Fex-3 (**24**) was attributed to its higher lipophilicity (cLogP = 7.42) compared to Fex (**20**) (cLogP = 7.01) [34]. The authors hypothesized that Fex-3 (**24**) will have poor solubility and will accumulate in the intestinal epithelial cells, thereby becoming selective to the intestine only. When they tried to test this hypothesis by administering Fex-3 (**24**) intraperitoneally (i.p.), the ligand precipitated and adhered to the intestine walls [34]. To further explore the effect of Fex-3 (**24**) in vivo, it was administered to mice orally at a dose of 100 mg/kg for 5 days. The results showed a significant increase in the expression of SHP in the ileum, while no such increase was observed in the liver, kidney, or other parts of the intestine. Additionally, Fex-3 (**24**) induced FXR downstream genes SHP and BSEP significantly compared to Fex (**20**) while suppressing the expression of CYP7A1 [34].

Molecular modeling of Fex-3 (**24**) using the X-ray cocrystal structure of Fex (**20**) with FXR (PDB ID: 1OSH) showed that both Fex (**20**) and Fex-3 (**24**) occupy the same cavity, while Fex-3 (**24**) binds deeper inside the ligand binding pocket. Experimental validation showed that Fex-3 (**24**) was two orders of magnitude higher in binding affinity (K_d_) than Fex (**20**) [34]. Further research is needed to determine the efficacy and safety of Fex-3 as it has not been studied in clinical trials.

**Figure 6:**
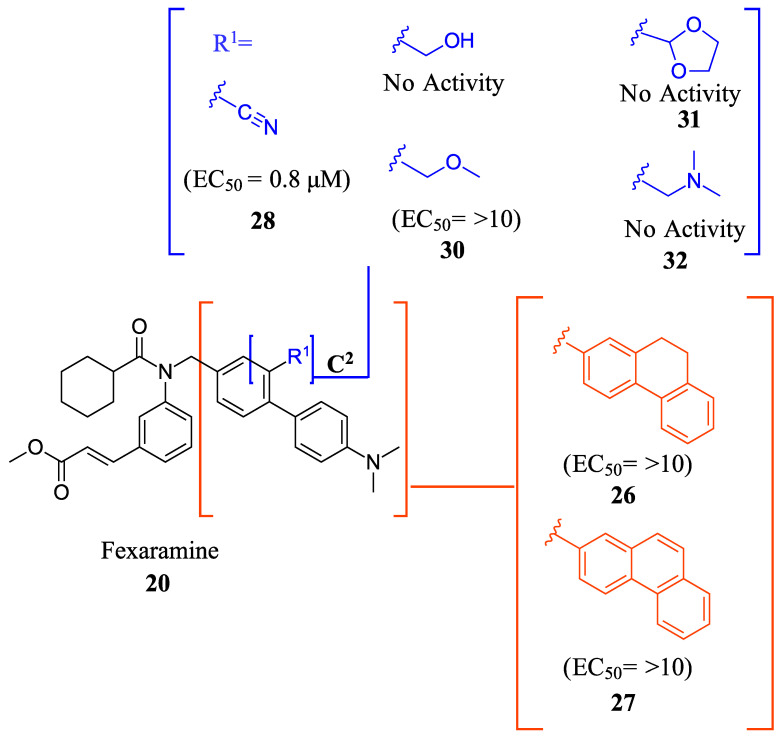
Development of compound **28** from Fex (**20**).

**Figure 7:**
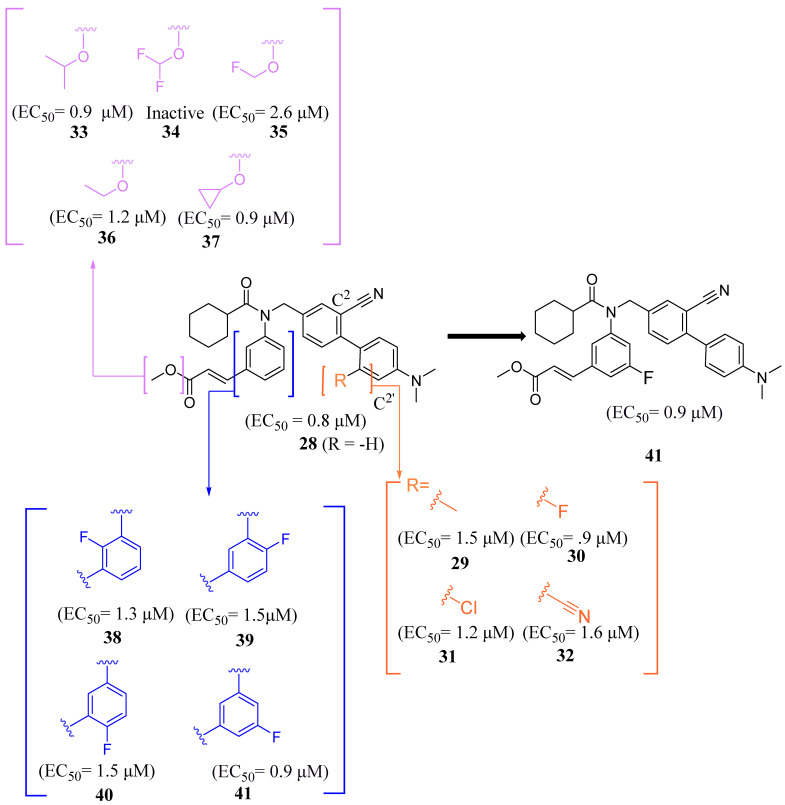
Development of compound **41** from **28**.

Compound **41** is a partial FXR agonist derived from the molecular structure Fex (**20**) (Figure 7). Compound **41** was developed by systematically optimizing different parts of Fex (**20**), starting with the synthesis of compound **28** (Figure 6). Initially, the flexible biphenyl ring in Fex (**20**) was replaced with various tricyclic ring structures to assess the binding pocket’s capability to accommodate a larger, constrained group. Unfortunately, this modification resulted in decreased activity, as observed in compounds **26** (EC_50_ >10 μM, *E_max_* = 14% at 3.3 μM) and **27** (EC_50_ > 10 μM, *E_max_* = 9% at 3.3 μM) (Figure 6) [35]. To examine if altering the biphenyl ring could improve hydrogen bonding with the LBD, five various substituents were added to C^2^. These compounds were tested for agonist activity in an NHR protein interaction assay compared to GW4064 (**1**) (EC_50_ = 0.4 μM, *E_max_* = 100% at 3.3 μM). The introduction of substituent groups such as methyl hydroxy, methyl ester, and dioxolane led to a loss of activity. However, when a cyano group was utilized, compound **28** was discovered, exhibiting enhanced potency and efficacy compared to other compounds in the series. Compound **28** demonstrated an EC_50_ of 0.8 μM and an *E_max_* of 24% (Figure 6) [35]. In order to assess the selectivity of compound **28**, it was tested against eighteen nuclear receptors at a concentration of 100 µM using a PathHunter NHR Protein Interaction Assay. The results showed that this compound did not activate any other receptors except for the desired one [35]. This promising outcome prompted further optimization of the molecule.

Next, the 4′-dimethylaminophenyl ring, aniline ring, and acrylic acid methyl ester moiety were studied using an NHR Protein Interaction Assay. The addition of small groups such as methyl, cyano, and fluorine to C^2^′ of the *p*-dimethylaminophenyl ring diminished activity. Modifications were then made to the acrylic acid methyl ester moiety by adding cyclopropyl, fluoromethyl, difluoro methyl, cyclopropyl, or ethyl instead of the methyl group [35]. Replacing the methyl ester with the aforementioned groups did not improve activity and, in some cases, diminished it. Out of all compounds with fluorine added to the aniline ring, **41** retained activity, with the fluorine in the C^5^´ position of the ring (EC_50_ = 0.9 μM, *E_max_* = 56%) (Figure 7) [35].

Binding of **41** was studied using the X-ray of Fex (**20**) bound with FXR (PDB ID: 1OSH). The biphenyl rings have hydrophobic interactions with Met269, Met294, Ile339, Phe340, Leu344, and Leu352 while the dimethylamine hydrophobically bonds with Leu344 [35]. Importantly, the C´^5^ fluorine of **41** occupies a pocket formed by Phe340, Leu352, Ile356, Met369, Phe370, and Tyr373, anchoring the ring in place. This allows for the methyl ester group to form a hydrogen bond with Asn287 while the amide carbonyl oxygen hydrogen bonds with His298, an interaction conserved from the FXR–Fex (**20**) crystal structure [35]. In comparison to the partial agonist Nidufexor (*E_max_* = 57%) and Fexaramine Fex (**20**) (*E_max_* = 40%), **41** showed a maximum efficacy of 53% [35]. It should be noted that this is the only article that describes Fex (**20**) as a partial agonist.

**Figure 8:**
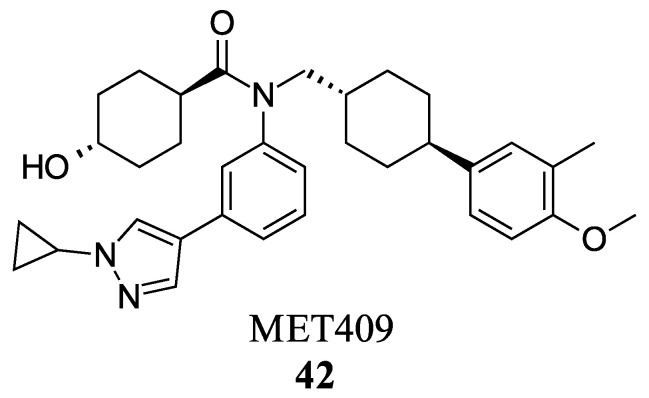
Structure of MET409 (**42**).

MET409 (**42**) is a Fex (**20**) derived FXR agonist developed by pharmaceutical company Metacrine for the treatment of NASH (Figure 8). MET409 is currently undergoing phase 2a clinical trials and has displayed favorable pharmacokinetic and pharmacodynamic properties. However, it is important to note that although some patents have described MET409 as being selectively targeted towards the intestines, these claims lack supporting data [36].

In a study involving male C57BL/6J mice fed a diet high in trans-fat, fructose, and cholesterol, the effects of MET409 (**42**) were investigated. The mice were administered MET409 (**42**) at various doses (1 mg/kg, 3 mg/kg, and 10 mg/kg) via oral gavage. The results demonstrated a dose-responsive improvement in the NAFLD activity score (NAS) upon treatment. The NAS improvements were reported as 42% at a dose of 1 mg/kg, 67% at 3 mg/kg, and 75% at 10 mg/kg [37]. Liver galectin-3 levels improved by 56–69% with increasing dosage. Fibrosis was assessed by total Col1a1 levels, which improved by 38–49% [37]. In clinical trials, doses of 80 mg or 50 mg of MET409 resulted in a 55% and 38% reduction in liver fat content, respectively [38]. ALT levels decreased by 25% and 28% for 80 mg and 50 mg, respectively, accompanied by a reduction in high-density lipoprotein cholesterol by 23.4% at 80 mg and 20.3% at 50 mg [38]. Studies have shown that the development of FXR agonist drugs can cause an increase in low-density lipoprotein cholesterol (LDL-C) and pruritus levels, which led to the termination of clinical trials in the past [38]. At a dosage of 80 mg, LDL-C levels increased by 23.7%, while at 50 mg, the increase was 6.8%. Pruritus was reported in 16% of patients who took 50 mg and 40% of patients who took 80 mg [38]. However, further evidence is needed to support the claim that MET409 (**42**) could be a safer FXR agonist at 50 mg due to its distinct pruritus and LDL-C profile, as well as its potential for intestinal selectivity.

**Figure 9:**
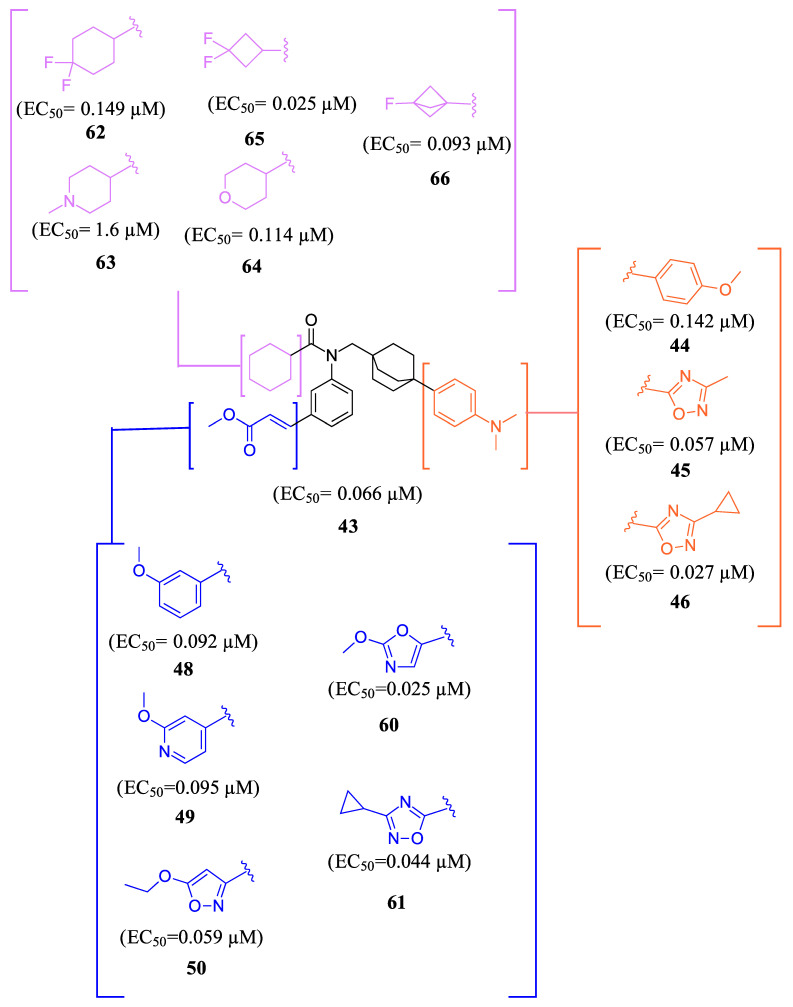
Development of compound **61** from **43**.

To develop an effective FXR agonist with reduced pruritus and elevated blood lipid levels, BMS-986318 (**74**) was designed (Table 1) [39]. Fex (**20**) was chosen as a starting point due to its unique co-crystal structure when bound to FXR. Optimization began with replacing the first ring in the biphenyl of Fex (**20**) with [2.2.2] bicyclooctane to make **43** (Figure 9) [39]. This was performed as molecular modeling revealed a large, hydrophobic, barrel-shaped pocket in Fex’s biphenyl region that could be targeted for improved binding. This modification led to a 20-fold increase in potency (EC_50_ = 0.066 µM) when compared to GW4064 (**1**). In hFXR-Gal4 reporter assays, compound **43** had a selective activation effect on IBABP, while BSEP remained unchanged. In Huh-7 liver cells, compound **43** was found to be completely inactive. This indicated that compound **43** was tissue-selective, as IBABP is predominantly in the intestines while BSEP is mainly expressed in the liver [39].

Further optimization was made to the aromatic amine as it has mutagenic and carcinogenic potential [39]. Replacing the amine with a methoxy group in **44** led to a reasonable improvement in potency (EC_50_ = 0.142 µM), indicating that the amine group is not necessary to retain activity (Figure 9). Replacing the methoxy phenyl with a smaller heteroaromatic ring in **45** led to improved activity (EC_50_ = 0.057 µM) with a ClogP of 6.64. Activity was further improved in compound **46** with the addition of a cyclopropyl group to the ring (EC_50_ = 0.027 µM). It was necessary to replace the reactive α,β-unsaturated ester group to avoid potential idiosyncratic adverse effects [39]. Therefore, drug-like moieties were used to replace this group to improve the physicochemical properties and solubility. Various aryl and heteroaryl rings were tested, and it was discovered that the 5-member heteroaryl rings demonstrated the best activity (e.g., **61**). For instance, cyclopropyl oxadiazole **61** displayed good activity based on the Gal4 reporter assay, with an EC_50_ of 0.044 µM (Figure 9).

Studies on compound **61** showed that in both mice and humans, the cyclohexyl group was mainly oxidized during biotransformation. As a result, modifications were made to the cyclohexyl group (Figure 9) [39]. The new compounds are not only being evaluated for their activity in the hFXR-Gal4 reporter assay but also for their PXR and recombinant cytochrome P450 isoform inhibition and metabolic stability. These compounds showed single digit µM activity. To enhance the metabolic stability, the cyclohexyl group was replaced with a fluro[1.1.1]bicyclopentane to produce compound **66**. This substitution demonstrated relatively good potency (EC_50_ = 0.093 µM) and a significant improvement in stability compared to the cyclohexyl group (100% remaining after 10 min of incubation with 1 mg/mL) (Figure 9).

To enhance stability, several modifications were made to the oxadiazole ring. Substituting cyclopropyl with tert-butyl not only improved its potency in the Gal4 assay but also eliminated PXR transactivation and CYP inhibition. Although the isomeric oxadiazole compound **68** exhibited better potency (EC_50_ = 0.029 µM), it had a poor CYP inhibition profile. To address this, various groups were used to replace the cyclopropyl oxadiazole, but none of them seemed to improve CYP liability (Table 1). Fluorinated groups were then added to the tert-butyl group but did not help with CYP liability either. To extend polarity to the third arm of the compound, a polar metabolically stable replacement for the [1.1.1] bicyclopentane was explored. Modifications including trifluoromethoxy cyclobutane led to the identification of BMS-986318 (**74**), which had a reasonable CYP inhibition profile and no PXR transactivation potential (Table 1).

In a co-activator recruitment assay of hFGF19 and IBABP gene expression, BMS-986318 (**74**) exhibited increased potency and reduced efficacy when compared to GW4064 (**1**). In hepatocytes, BMS-986318 (**74**) displayed reduced activity demonstrating tissue-dependent activation. In an in vitro mouse bile duct ligation model, pharmacological evaluation of BMS-986318 (**74**) was conducted by oral administration (0.3, 1, 3, and 10 mg/kg day). In comparison to an isoxazole counterpart, this compound was discovered to induce liver FGF15 at a 50-fold lower level. However, it exhibited a similar extent of FGF15 induction in the ileum. This confirms the tissue-selective profile of BMS-986318 (**74**). Collagen deposition induced by liver fibrosis was lowered in a dose-dependent manner, indicating the antifibrotic effects of BMS-986318 (**74**).

LH10 (**82**) was designed by modifying two of the three main regions of Fex (**20**) (EC_50_ = 0.30 µM) to obtain higher activity (Table 2) [40]. While LH10 (**82**) showed higher activity overall (EC_50_ = 0.14 µM), it is important to note that this drug has not been tested for intestinal selectivity. To develop LH10 (**82**), modifications were made to the biphenyl moiety of Fex (**20**) by incorporating hydrophilic groups. The aim of these alterations was to enhance the compound’s draggability [40]. However, it was observed that these modifications resulted in a significant decrease in activity (i.e., **75**–**77**) (Table 2) [40].

In an effort to enhance the compound, improvements were made to the methyl cinnamate located in region 2 of Fex (**20**). Benzylic acid groups were introduced in order to potentially form an ionic bond with the basic Arg355 residue [40]. However, the compounds **78** (with an EC_50_ > 10 µM) and **79** (with an EC_50_ of 3.87 µM) did not exhibit better performance compared to Fex (**20**) [40]. Nonetheless, they demonstrated superior activity compared to the corresponding methyl esters, **80** (with an EC_50_ of 9.58 µM) and **81** (with an EC_50_ > 10 µM) [40].

LH10 (**82**) (EC_50_ = 0.14 µM), a compound derived from Fex (**20**), showed improved activity by replacing the second phenyl ring of region 1 in **20** with benzoheterocycles and substituting region 2 of **20** with cyclopropyl pyrazolyl. Additionally, the introduction of a benzofuran group to region 1 further contributed to the enhanced potency of LH10 (**82**) (Table 2) [40]. When benzofuran was replaced with different benzoheterocycles, a notable decrease in activity was observed. For instance, compounds **83** (with an EC_50_ of 0.25 µM) and **85** (with an EC_50_ of 2.81 µM) exhibited reduced potency. Similarly, substituting the cyclopropyl pyrazolyl in region 2 resulted in diminished activity, as seen with compound **84** (with an EC_50_ of 0.57 µM) (Table 2) [40]. Alterations made to region 3 also led to a decline in activity, as evidenced by compound **86** (with an EC_50_ of 0.45 µM) [40].

LH10 (**82**) was further evaluated for target specificity against LXRα, LXRβ, THRβ, PPARα, PPARγ, and PPARδ and showed excellent selectivity toward FXR, making it a candidate for further studies [40]. LH10 (**82**) was then virtually docked into a crystal structure of FXR and compared to Fex (**20**). It was found that LH10 (**82**) occupies the same pocket as Fex (**20**) but has a much higher affinity score compared to Fex (**20**). The cyclohexanecarboxamide of LH10 (**82**) forms a hydrogen bond interaction with His298. Additionally, the two benzene rings of LH10 (**82**) exhibit σ–π interactions with Ile356 and Leu291 [40].

To evaluate LH10 (**82**) in ANIT-induced cholestatic liver disease models, mice were pretreated with an oral dose of LH10 (**82**) (20 mg/kg) followed by an oral administration of ANIT (50 mg/kg). The physiological condition of the mice treated with LH10 (**82**) demonstrated improved levels of AST, ALT, lactate dehydrogenase (LDH), alkaline phosphatase (ALP), and total bile acid (TBA) [40]. These findings suggest that LH10 (**82**) had a positive effect on liver injury and cholestasis in the mice.

Further experiments were conducted using ANIT-induced cholestatic liver disease models in C57BL/6 mice, which are known for their different sensitivity. Comparisons were made between LH10 (**82**) and OCA (**7**) in this model, and LH10 (**82**) outperformed OCA (**7**) [40]. OCA (**7**) treatment resulted in hepatic necrosis in liver cells, while LH10 (**82**) treatment did not exhibit such effects. Both treatment groups showed decreased levels of ALP, TBA, and TBIL; however, LH10 (**82**) demonstrated a more significant reduction in TBIL compared to OCA (**7**). Moreover, in APAP-induced acute liver injury models, histological analysis revealed improvements in bleeding points, degeneration, and necrosis of liver cells upon treatment with LH10 (**82**). Additionally, LH10 (**82**) led to decreased levels of AST, ALT, LDH, and ALP, which are indicative of liver injury [40].

In a NASH mice model induced by a high-fat Western diet (WD) and carbon tetrachloride (CCl_4_), treatment with LH10 (**82**) or OCA (**7**) (administered orally at a dose of 20 mg/kg) for one month resulted in significant improvements. Both LH10 (**82**) and OCA (**7**) demonstrated considerable amelioration of NASH characteristics, including steatosis, ballooning, and inflammatory infiltration [40]. Fibrosis, a hallmark of NASH, is associated with elevated levels of transforming growth factor-beta l (TGF-β1) and hydroxyproline (HYP). Treatment with LH10 (**82**) and OCA (**7**) led to a notable reduction in the expression of TGF-β1 and HYP, indicating the potential attenuation of liver fibrosis [40].

In terms of lipid metabolism, LH10 (**82**) exhibited regulatory effects by downregulating angiopoietin-like protein 3 (ANGPTL3), SREBP-1c, and enzymes ACC and FAS [40]. Simultaneously, LH10 (**82**) upregulated the expression of APOC2 and lipoprotein lipase (LPL), resulting in the limitation of lipid production in the liver. Furthermore, LH10 (**82**) demonstrated improved inflammation parameters by reducing the expression of TNF-α, IL-1β, IL-6, and monocyte chemotactic protein-1 (MCP-1) [40]. It also exhibited a beneficial effect on liver oxidative stress by increasing the levels of superoxide dismutase (SOD) and glutathione peroxidase (GSH-px). Overall, LH10 (**82**) demonstrated significant improvements in NASH-related parameters, including fibrosis, lipid metabolism, inflammation, oxidative stress, and FXR activation. Further research is warranted to investigate potential side effects associated with the administration of LH10 (**82**) [40].

#### 2.2.2. Miscellaneous

**Figure 10:**
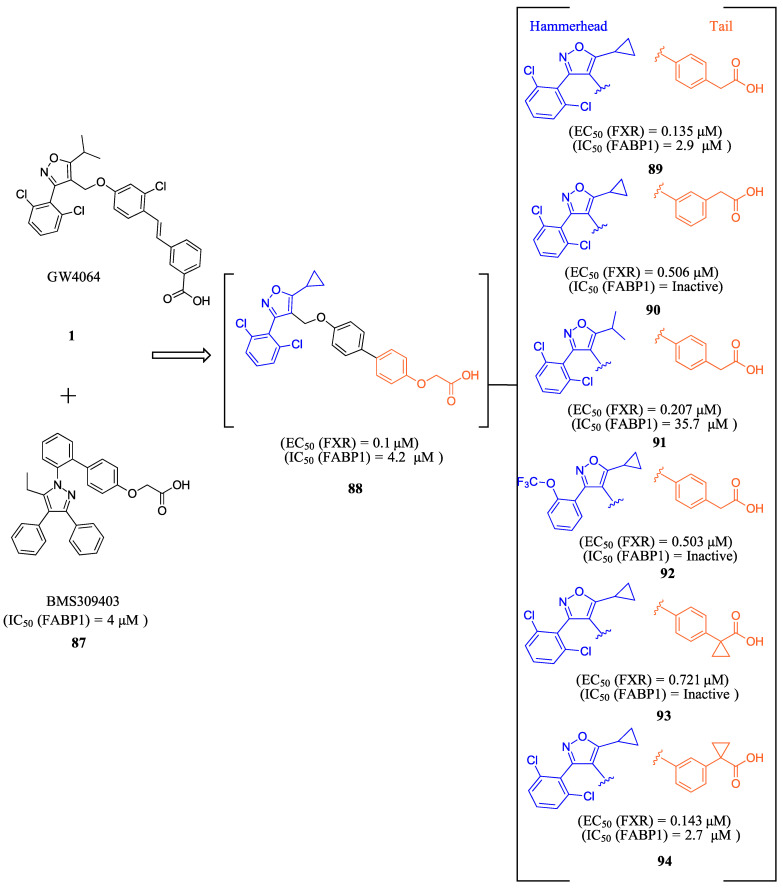
Development of ZLY28 (**94**).

ZLY28 (**94**) was discovered while pursuing an FXR-FABP1 dual modulator by improving on the GW4064 (**1**) scaffold (Figure 10). GW4064 (**1**) was chosen due to its ability to inhibit FABP1 in comparison to other well-known FXR agonists (EC_50_ (FXR) = 0.065 μM; IC_50_ (FABP1) = 35.2 μM) [41]. In hopes of mitigating the undesirable properties of GW4064 (**1**), the questionable stilbene moiety was replaced with a biphenyl (**88**) as seen in an FABP4 inhibitor BMS309403 (**87**) (Figure 10) [42]. Initially, it was observed that the direct hybrid analog (**88**) of GW4064 (**1**) and BMS309403 (**87**) improved FABP1 inhibition compared to GW4064 (**1**), indicating that the biphenyl moiety would help induce FABP1 activity. Replacing the phenoxy acetic acid with phenylacetic acid afforded compound **89** (EC_50_ (FXR) = 0.135 μM; IC_50_ (FABP1) = 2.9 μM) (Figure 10) [41]. This newly developed compound demonstrated enhanced activation of FXR in cell-based assays. However, it was much weaker than the direct hybrids in the FRET assay. Notably, relocating the acetic acid group to the meta position resulted in complete abolition of its activity (i.e., **90**). Modifying the substituents on the phenyl ring or the cyclopropyl, derived from the hammerhead structure of compound 1, also led to decreased activity (i.e., **91–92**) (Figure 10) [41].

Further improvements were then made to compound **89** by constraining the flexibility of the phenylacetic acid. Moving the acetic acid group to the meta position and introducing a cyclopropyl group to the α-position of the acetic acid resulted in similar outcomes to those observed in compound **89**, as demonstrated by compound ZLY28 (**94**) (EC_50_ (FXR) = 0.143 μM; IC_50_ (FABP1) = 2.7 μM) (Figure 10) [41]. In in vitro liver microsomal metabolic studies, ZLY28 (**94**) and **89** exhibited good stability, with over 50% of the original compound remaining after one hour. In a selectivity study, ZLY28 (**94**) and **89** were tested against FABP3/4; free fatty acid receptor 1; TGR5; and nuclear receptors including LXRα/β, PPARα/β/γ, THRβ, RXRα, RARα/γ, VDR, ERα/β, PR, and GR. The study found that ZLY28 (**94**) and **89** are highly selective dual modulators of FXR/FABP1 while moderately inhibiting FABP4 [41].

The analysis of tissue distribution indicated that ZLY28 (**94**), although structurally similar to **89**, showed predominant distribution in the ileum. Furthermore, it was found to upregulate ileal SHP, FGF15, and OSTβ. The release of FGF15 caused a decrease in the expression of CYP7A1 in the liver. Compound **89** was mainly distributed to plasma and liver and upregulated downstream SHP, BSEP, and OSTβ.

The effects of ZLY (**94**) and **89** were compared with OCA (**7**) in WD and CCl_4_ NASH models. In the WD model, all three drugs demonstrated positive effects on NASH, but only ZLY28 (**94**) resulted in a reduction in liver-to-body weight ratio. Both OCA (**7**) and ZLY28 (**94**) showed improvements in hepatic steatosis, lobular inflammation, and ballooning. In terms of hepatic lipid levels, ZLY28 (**94**) was more effective than OCA (**7**) in reducing total cholesterol levels and non-esterified fatty acids in the feces. Therefore, ZLY28 (**94**) shows promise as a potential leading agonist that could be considered for future clinical trials [41]. In CCl_4_ model, ZLY28 (**94**) has been shown to downregulate genes related to lipogenesis, such as SREBP-1c and acetyl-CoA carboxylase 1, while at the same time upregulating genes associated with lipolysis, such as Apo C-II and lipoprotein lipase. This is achieved by targeting both FXR and FABP1 [41]. In general, **89** and ZLY28 (**94**) improved hepatic lipid homeostasis by synergistically inhibiting lipogenesis and promoting lipolysis. ZLY28 (**94**) also significantly reduced the expression of inflammatory factors such as IL-1β, IL-6, and MCP-1 while decreasing expression of IL-1β, IL-6, TNF-α, and F4/80 [41].

In liver fibrosis models accelerated by CCl_4_, both **89** and ZLY28 (**94**) have been found to reduce fibrosis. This is achieved by inhibiting the expression of the profibrotic factor TGF-β and α-SMA, which in turn inhibit hepatic stellate cells. ZLY28 (**94**) was found to be more effective than Fex (**20**) in increasing oxidative-stress-reducing antioxidant enzymes such as superoxide dismutase and glutathione peroxidase. This indicates that ZLY28 (**94**) may be a better option for reducing oxidative stress. ZLY28 (**94**) was tested for toxicity in mice at a dosage of 500 mg/kg/day (p.o.) for 14 days and displayed no acute toxicity. Overall, ZLY28 (**94**) exhibits greater anti-NASH effects than **89** through dual modulation of FABP1 and FXR. ZLY28 (**94**) has been shown to activate intestinal FGF15, which regulates lipid metabolism inflammation, fibrosis, and oxidative stress while maintaining an acceptable safety profile [41].

**Figure 11:**
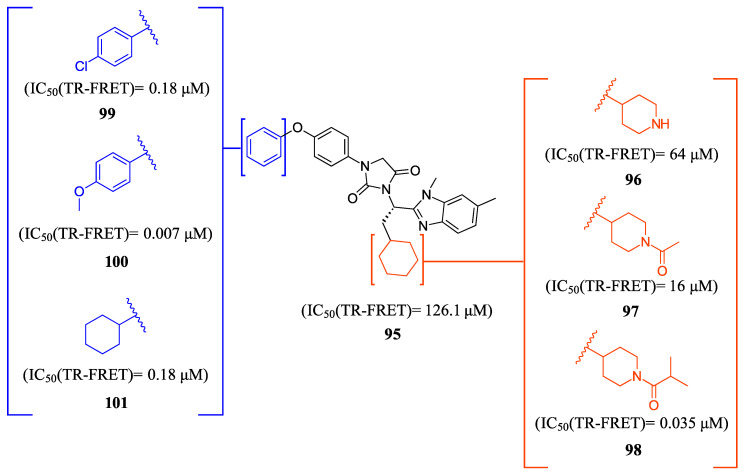
Development of compound **100**.

**Figure 12:**
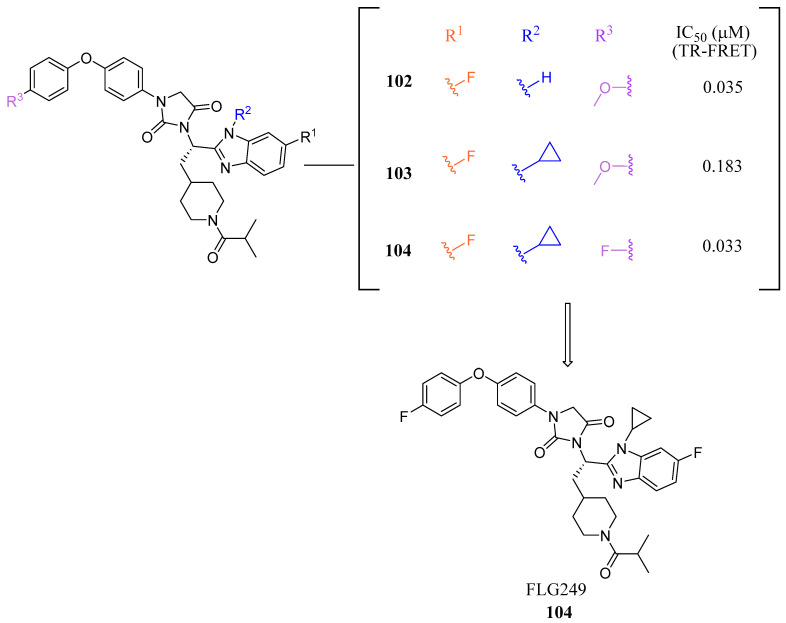
Development of FLG249 (**104**).

FLG249 (**104**) was developed as a potent FXR antagonist through optimization of the novel chemotype **95** (IC_50_ = 126.1 μM) (Figure 11), as shown in (Figure 12) [43]. In the FXR TR-FRET binding assay, there were notable improvements when replacing cyclohexane in **95** with piperidine substituted at the nitrogen atom with a large isobutyryl group, resulting in compound **98** (IC_50_ = 0.035 μM) (Figure 11). In virtual docking studies, **98** was found to create hydrogen bonding with His298 [43]. A comprehensive SAR analysis that studied each of the seven binding regions of **98** led to more important information. Firstly, the *S*-enantiomer of the isobutyryl piperidine is a necessary pharmacophore for antagonistic activity, and the *R*-enantiomer loses activity [44]. Next, the only known alterations to improve activity are made to the external phenoxy ring. Compound **100** was of great interest, as adding a para-phenoxy group led to nanomolar activity (IC_50_ = 0.007 ± 0.002 μM) (Figure 11) [44]. In order to better understand the PK profile and tissue distribution, a 6 h rat PK study was conducted comparing the effects of **100** (administered intravenously at a dosage of 1 mg/kg in 10% 2-hydroxypropyl-cyclodextrin) and **98** (administered orally at a dosage of 30 mg/kg in 40% HP-β-CD) [44]. Both compounds **98** and **100** showed modest half-lives and a high volume of distribution when given intravenously. Additionally, they exhibited a low ratio of urinary excretion, which suggests that some metabolite breakdown may have occurred.

Compound **100** exhibited a higher bioavailability (%F) of 17.99 ± 3.52, in contrast to 2.76 ± 0.31 observed for compound **98**. Furthermore, the study examined the plasma and tissue concentrations of compounds **98** and **100** six hours after oral administration. The result showed that compound **100** had significantly higher concentrations in target tissues such as the plasma, liver, and ileum when compared to compound **98**. Notably, the concentrations of compound **100** were 15 times higher in the liver and 13 times higher in the ileum compared to plasma [44]. Compound **100** outperforms compound **98** by demonstrating superior PK profiles and distributions in liver and ileum while retaining its high potency as a specific FXR antagonist.

In mouse liver microsomes (MLM), it was observed that compound **100** exhibited low metabolic stability, with only 2% of the original molecule remaining after 30 min [45]. Modifications were made to improve its metabolic stability by implementing less metabolically susceptible moieties (Figure 12) [45]. Replacing the methyl group of the benzimidazole with fluorine led to the identification of compound **102** (IC_50_ = 0.035 µM), which exhibited a similar potency to compound **100** in the TR-FRET bioassay (Figure 12). The benzimidazole ring was further modified by replacing the *N*-Me with a cyclopropyl group to produce compound **103**, which was less potent than **102**. Replacing the methoxy group in the phenoxy moiety with fluorine resulted in the discovery of FLG249 (**104**) (Figure 12). This compound showed improved metabolic stability and activity. The results showed that 98% of compound **100** was metabolized in 30 min. However, only 57% of compound **103** and less than 50% of FLG249 (**104**) were metabolized. This indicates that the substitution of fluorine on the phenoxy ring hinders the oxidation of the benzene ring, making it more stable in comparison to compounds **102** and **103**.

Pharmacokinetic studies were conducted on rats to assess the efficacy of FLG249 (**104**). The drug was administered in two ways: intravenous (IV) injection of 1 mg/kg of the drug in 10% 2-hydroxypropyl-β-cyclodextrin and oral administration of 30 mg/kg of the drug in 40% HP-β-CD. The results showed that when administered orally, FLG249 (**104**) showed significantly higher AUC, C_max_, and bioavailability compared to **103** [45]. Importantly, FLG249 (**104**) had a tendency to accumulate in the ileum (116.45 ± 41.65 μg/g) six hours after oral administration, which was three times higher than the liver (38.42 ± 1.95 μg/g) and 46 times higher than the plasma [45]. FLG249 (**104**) was tested on male C57BL/6N mice to determine its effect on the regulation of target genes FGF15, ASBT, and SHP, which are expressed in the intestine. The mice were given the compound orally once a day for 7 days at doses of 10 and 30 mg/kg. The results showed that the antagonist FLG249 (**104**) significantly reduced SHP and FGF15 in the ileum, while ASBT was slightly increased with little dosage-dependent effect [45]. None of the target genes in the liver were affected by FLG249 (**104**), as expected. Finally, FLG249 (**104**) was tested on nine other nuclear receptors to assess its selectivity and proved to be an FXR selective antagonist [45].

## 3. Discussion

Substantial structural modifications were implemented across the three scaffolds described in this review, all of which exhibit pronounced selectivity for the farnesoid X receptor (FXR), with many also demonstrating intestinal selectivity. These scaffolds encompass steroidal ligands that retain the core bile acid structure of CDCA. Among them, antagonists such as GUDCA (**5**), Gly-MCA (**6**), and F6 (**19**) incorporate a central amide within the modified tail of the bile acid scaffold, a feature akin to that of T-β-MCA, a well-recognized FXR antagonist.

In contrast, agonists preserve the bile acid tail structure and enhance potency through structural additions—notably, the inclusion of an ethyl group at C6 and a hydroxy group at C11β. These modifications not only maintain but also improve efficacy over the endogenous ligand CDCA, thereby optimizing the therapeutic potential of these molecules in modulating FXR activity. Such strategic enhancements underscore the ongoing efforts to refine the molecular architecture of FXR ligands to achieve superior therapeutic outcomes.

Compounds featuring the core scaffold of Fex (**20**) are distinguished by their FXR-selectivity, intestinal restriction, and efficacy, rendering this scaffold a compelling candidate for further investigation in medicinal chemistry research. The core amide configuration within Fex (**20**) is preserved across all its derivatives, with the cyclohexyl ring typically maintained or subjected to minimal modifications, such as the incorporation of a hydroxyl group. An exception is noted in the agonist Fex-3 (**24**), where the substitution of a larger naphthalene ring coupled with a methylamine group enhances activity. The biphenyl segment of Fex (**20**) consistently maintains its structural integrity and length across modifications.

Alterations that increase chain length or introduce large, conformationally restricted ring structures typically diminish biological activity, as evidenced by derivatives such as **22**, **26**, and **27**. Conversely, incorporating non-aromatic, bicyclic, or small fused ring systems, including five-member heterocyclic rings, has been demonstrated to enhance activity, as seen in derivatives **42**, **74**, and **82**. The benzene ring directly linked to the nitrogen of the central amide is consistently retained across all derivatives, though minor substituent additions have been employed to augment activity. Furthermore, the acrylic acid methyl ester in this segment can be substituted with a heterocyclic or benzene ring, as demonstrated in derivatives **42**, **74**, and **84**; however, modifications to the ester group, when retained, generally reduce efficacy (e.g., **33–37**).

Additionally, derivative MET495 (**42**) exemplifies the potential for introducing specific stereochemistry into the Fex scaffold, which not only improves the therapeutic activity but also enhances the safety profile for clinical evaluation.

Among the non-steroidal ligands evaluated, the agonist ZLY28 (**94**) retains the essential head group of GW4064 (**1**), maintaining its activity while enhancing the molecule’s properties through the integration of a biphenyl moiety, a structural element inspired by BMS309403 (**87**). Although this modification did not elevate the activity of GW4064, ZLY28 (**94**) demonstrated superior performance compared to the well-known steroidal agonist OCA, significantly enhancing GW4064’s stability and positioning it as a more promising drug candidate.

The antagonist FLG249 (**104**) is derived from an entirely novel chemotype. Within this core structure, the central hydantoin is preserved, and the *S*-enantiomer of the isobutyryl piperidine is crucial for activity, as the *R*-enantiomer results in a loss of efficacy. Additionally, the incorporation of a fluorine atom in the phenoxy benzene group is essential for the stability of the compound, contrasting with the instability of the methoxy derivative (**100**). Remarkably, the addition of fluorine to the benzimidazole ring significantly enhances the activity of the ligand.

These structural insights provide valuable guidance for future researchers aiming to make informed modifications to these scaffolds, particularly in the design of intestinally restricted FXR ligands, fostering advancements in therapeutic agent development. 

Understanding the properties of a compound can make it easier to ensure it has optimal properties for a desired effect. Lipinski’s rule of five has long been a guideline for how to achieve oral bioavailability. Further studies were by Varma et al. conducted to determine what factors impact intestinal absorption. They found that increasing molecular weight, especially above 500, limits the capability of the molecule to pass the lipid membrane of the intestine [46]; further, a high hydrogen bond count (HBA + HBD > 9) is energetically unfavorable for membrane permeability [46].

Intestinally restricted drugs are sought-after FXR ligands due to their ability to minimize undesirable side effects. Drugs can achieve intestinal restriction by designing compounds that lack the requisite characteristics for intestinal permeability. It is notable that all known intestinally restricted FXR modulators possess a molecular weight of approximately 500 or greater (Table 3) and exhibit a high hydrogen bond count exceeding nine (Table 4). Previous research has established that low aqueous solubility can contribute to intestinal selectivity, as a drug must dissolve to be absorbed [47].

The program Qikprop, which predicts properties by comparing them to 95% of known drugs, was utilized to assess compounds described in this review and determine predictive properties that could aid in designing intestinally restricted FXR ligands [48]. All examined compounds exhibited low aqueous solubility, with non-steroidal ligands displaying exceptionally low solubility values below −9, compared to the typical range of −0.5 to −6.5. This finding underscores that very low aqueous solubility, around −9, is a critical factor to consider in the design of intestinally restricted FXR ligands.

Furthermore, Qikprop’s predictions of human oral absorption, based on a quantitative multiple linear regression model, indicated that all compounds listed had medium to high oral absorption. This outcome is intriguing, given that nearly all these compounds are known to be intestinally restricted. Conversely, qualitative predictions of human oral absorption based on factors such as LogP, number of rotatable bonds, solubility, and cellular permeability generally rated most compounds at 1, indicating low absorption (Table 4). This suggests that qualitative predictions are more suitable for designing intestinally restricted FXR ligands.

## 4. Conclusions

FXR holds promise as a target for metabolic disease treatment; however, many ligands face clinical trial setbacks due to side effects. One potential solution is to design FXR modulators restricted to the intestines, as liver activation can induce side effects. This review explores all known intestinally restricted FXR modulators and their optimization. Deviating from Lipinski’s rule of five is often a path to achieve intestinal selectivity [47]. The intestinally selective compounds discussed in this review typically have a high molecular weight (400–650 g/mol), exceeding Lipinski’s rule. The low permeability of these compounds could be due to tight junctions in the intestinal lining preventing larger compounds from absorbing [49]. Additionally, all compounds have at least five hydrogen bond acceptors, although their effect on permeability is lower [50]. High lipophilicity is associated with low solubility and intestinal selectivity. Nonsteroidal compounds described here exhibit high CLogP values (5.75–7.5), contributing to their intestinal selectivity [47].

Finally, properties of the ligands were calculated with Qikprop, a program that predicts properties by comparing them to 95% of known drugs [48]. It was established that the number of hydrogen bonds, aqueous solubility, and qualitative human oral absorption are aligned with low intestinal absorption. These parameters can serve as indicators for designing intestinally selective FXR ligands, providing a framework for identifying the necessary properties to achieve targeted drug selectivity within the gastrointestinal tract.

In conclusion, this review sheds light on the structural and chemical characteristics that could serve as a roadmap for the design and development of novel, intestinally restricted FXR modulators. The similarity in the structural features of the ligands discussed in this review highlights the importance of certain chemical moieties for achieving intestinal selectivity. The insights provided in this review could aid researchers in the design of more effective and safer ligands that target FXR for the treatment of metabolic disorders.

## Figures and Tables

**Table 1:** Development of BMS-986318 (**74**).

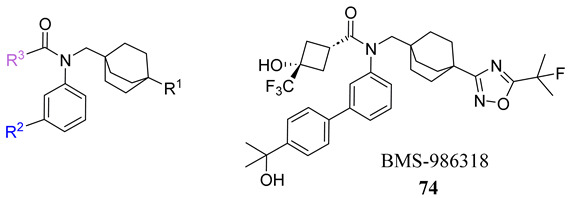				
**Comp.**	**R^1^**	**R^2^**	**R^3^**	**EC_50_ (µM)**
**67**	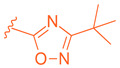	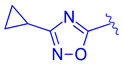	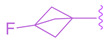	0.70
**68**	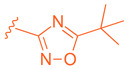	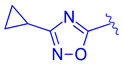	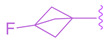	0.029
**69**	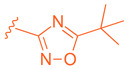	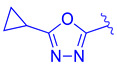	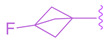	0.045
**70**	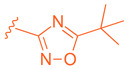	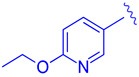	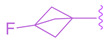	0.104
**71**	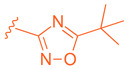	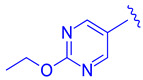	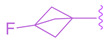	0.041
**72**	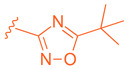	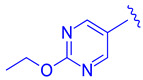	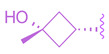	0.111
**73**	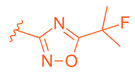	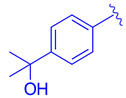	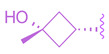	0.025
**74**	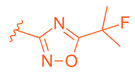	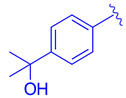	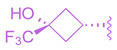	0.034

**Table 2:** Development of LH10 (**82**).

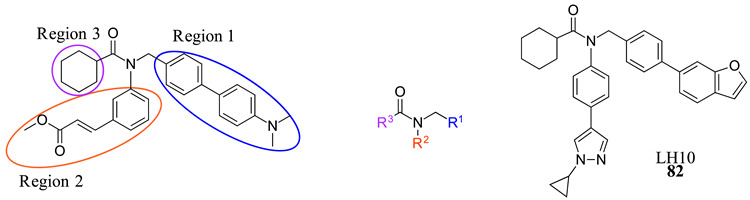				
**Comp.**	**R^1^**	**R^2^**	**R^3^**	**EC_50_ (µM)**
**75**	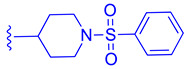	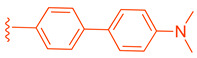	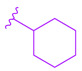	4.98
**76**	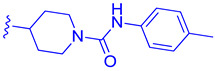	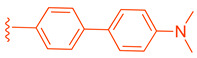	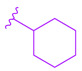	3.81
**77**	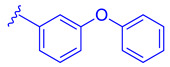	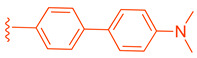	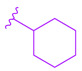	1.07
**78**	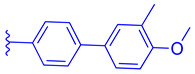	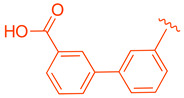	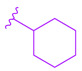	>10
**79**	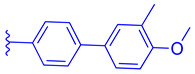	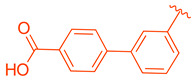	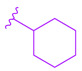	3.87
**80**	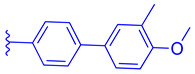	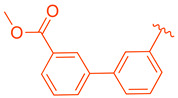	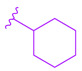	9.58
**81**	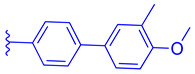	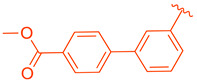	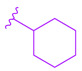	>10
**82**	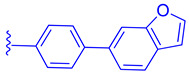	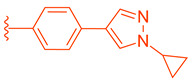	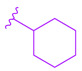	0.14
**83**	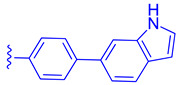	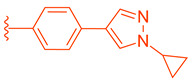	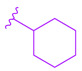	0.28
**84**	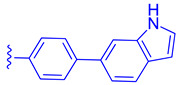	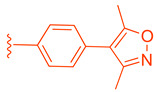	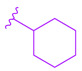	0.57
**85**	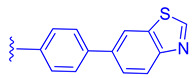	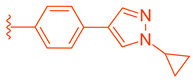	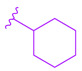	2.81
**86**	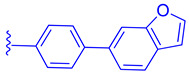	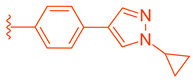	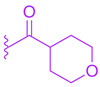	0.45

**Table 3:** Physical and chemical properties of intestinally selective FXR ligands.

Comp.	Systemic Effects	Gene Effects	EC_50_ μM	IC_50_ μM	CLogp	pKa (Acidic)	MW
**GUDCA** **(5)**	Increased metabolic rate and GLP1 production	Decreased FGF19 and SHP	-	77.2	2.61	3.77	449.31
**Gly-MCA (6)**	Reduced ceramide levels	Decreased SHP and FGF15	-	N/A	4.2	3,1	465.62
**F6 (19)**	Reduced weight gain Improved insulin sensitivity, glucose tolerance, and reduced ceramide levels	Increased FGF15, OSTα, and SHP Lowered ALT, AST, and TG	-	2.1	7.47	4.11	585.87
**TC-100 (8)**	N/A	Increased CYP7A1, SHP, OSTα, FGF15, ANG1, and BSEP	0.14	-	3.12	4.48	436.63
**Fex** **(20)**	Increased energy expenditure and weight loss Lowered glucose, insulin levels, Improved ethanol induced hepatic inflammation	SHP, MRP-2, BSEP; PLTP, intestinal Nr0b2, and FGF15	0.025	-	7.21	4.82	496.65
**Fex-D** **(23)**	Decreased ILC3, total intestinal serum BA	SHP, FGF15, OSTα, cytokine genes, and fibrotic genes	-	-	7.21	4.82	496.65
**Fex-3 (24)**	Increased intestinal selectivity	BSEP, CYP7A1, FGF15, and SHP	-	-	7.41	4.96	569.71
**41**	Reduced liver fibrosis and dyslipidemia effects	Increased IBABP, FGF15, SHP, and OSTα	0.9	-	7.21	4.60	539.65
**Met409 (42)**	Lowered liver fat content, liver fibrosis, and HDL in NASH models	Improved Col1a1 and Liver galectin-3 levels Decreased ALT	0.016	-	7.0	16.1	541.732
**BMS-986318 (74)**	Lower liver fibrosis	Increased FGF15/19, IBABP	0.034	-	6.55	11.23	643.72
**LH10 (82)**	Improved steatosis, ballooning, and inflammatory in NASH models	Improved ALT, AST, ALP, LDH, and TBA Decreased TBIL	0.14	-	7.7	2.3 (basic)	515.657
**ZLY28 (94)**	Reduced liver body weight ratio Lowered hepatic lipid levels Improved hepatic steatosis, lobular inflammation, and ballooning	SHP, FGF15, and OSTβ	0.143	(FABP1) 2.7	7.4	3.4	520.41
**FLG249 (104)**	N/A	Decreased FGF15 and SHP Increased ASBT	N/A	0.033	5.75	12.33	641.72

**Table 4:** ADME properties of intestinally selective FXR ligands.

Comp.	SASA	HBA	HBD	QPlogPo/w	QPlogS	Human Oral Absorption	% Human Oral Absorption	Rule of 5 Violations
**GUDCA (5)**	746.058	9	4	2.392	−4.085	2	62.258	0
**Gly-MCA (6)**	749.774	7	3	3.192	−4.884	2	56.576	0
**F6 (19)**	923.429	6	1	7.246	−9.174	1	75.705	2
**TC-100 (8)**	674.678	7	4	3.593	−4.480	3	77.265	0
**Fex (20)**	925.627	6	0	7.120	−9.151	1	100	1
**Fex-D (23)**	924.731	6	0	7.125	−9.134	1	100	1
**Fex-3 (24)**	1019.959	7	1	7.823	−10.537	1	100	2
**41**	909.912	7	0	6.472	−9.109	1	89.651	2
**Met409 (42)**	974.504	6	1	7.304	−10.380	1	100	2
**BMS-986318 (74)**	1001.255	7	2	7.966	−10.923	1	95.677	2
**LH10 (82)**	896.199	5	0	7.97	−9.768	1	100	2
**ZLY28 (94)**	825.370	4	1	7.474	−9.362	1	83.381	2
**FLG249**	1003.014	8	0	14.075	−9.226	1	100	2

SASA = total solvent exposed surface area; QPlogPo/w = Predicted octanol/water partition coefficient—(−2.0–6.5); QPlogS = Predicted aqueous solubility—Normal range from (−6.5–0.5); HumanOralAbsorption = Predicted qualitative human oral absorption (1 (low)–3 (high)); PercentHumanOralAbsorption = Predicted human oral absorption on 0 to 100% scale.

## Data Availability

No new data were created.
